# *In vivo* Mechanisms of Antibody-Mediated Neurological Disorders: Animal Models and Potential Implications

**DOI:** 10.3389/fneur.2019.01394

**Published:** 2020-02-05

**Authors:** Maria Pia Giannoccaro, Sukhvir K. Wright, Angela Vincent

**Affiliations:** ^1^Department of Biomedical and Neuromotor Sciences, University of Bologna and IRCCS Istituto delle Scienze Neurologiche di Bologna, Bologna, Italy; ^2^Nuffield Department of Clinical Neurosciences, University of Oxford, Oxford, United Kingdom; ^3^School of Life and Health Sciences & Aston Neuroscience Institute, Aston University, Birmingham, United Kingdom; ^4^Department of Neurology, Birmingham Children's Hospital, Birmingham, United Kingdom

**Keywords:** animal models, neuronal surface antibodies, passive transfer, maternal transfer, active immunization

## Abstract

Over the last two decades, the discovery of antibodies directed against neuronal surface antigens (NSA-Abs) in patients with different forms of encephalitis has provided a basis for immunotherapies in previously undefined disorders. Nevertheless, despite the circumstantial clinical evidence of the pathogenic role of these antibodies in classical autoimmune encephalitis, specific criteria need to be applied in order to establish the autoimmune nature of a disease. A growing number of studies have begun to provide proof of the pathogenicity of NSA-Abs and insights into their pathogenic mechanisms through passive transfer or, more rarely, through active immunization animal models. Moreover, the increasing evidence that NSA-Abs in the maternal circulation can reach the fetal brain parenchyma during gestation, causing long-term effects, has led to models of antibody-induced neurodevelopmental disorders. This review summarizes different methodological approaches and the results of the animal models of *N*-methyl-d-aspartate receptor (NMDAR), leucine-rich glioma-inactivated 1 (LGI1), contactin-associated protein 2 (CASPR2), and α-amino-3-hydroxy-5-methyl-4-isoxazolepropionic acid receptor (AMPAR) antibody-mediated disorders and discuss the results and the limitations. We also summarize recent experiments that demonstrate that maternal antibodies to NMDAR and CASPR2 can alter development in the offspring with potential lifelong susceptibility to neurological or psychiatric disorders.

## Introduction

**Figure 1:**
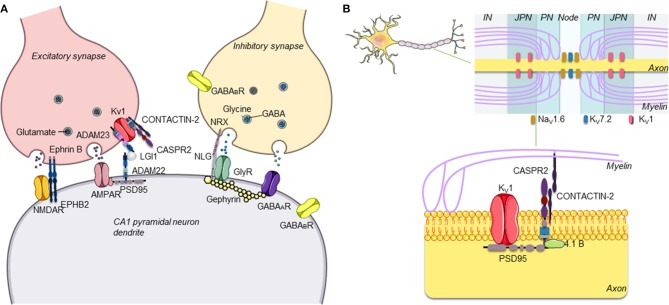
**(A)** Schematic representation of central excitatory and inhibitory synapses and main antibody targets. The proteins targeted by antibodies associated with autoimmune encephalitis are proteins expressed on the neuronal surface, often at both presynaptic and postsynaptic levels on inhibitory (GABAergic) and/or excitatory (glutamatergic) neurons in the central nervous system (CNS). **(B)** Schematic representation of CASPR2. CASPR2 localizes at the juxtaparanode of myelinated axons. CASPR2 binds to contactin-2/TAG-1 via its extracellular domain and links to PDZ-binding proteins and to the cytoskeleton via protein 4.1B, stabilizing Kv1 channels [adapted with permission from Giannoccaro et al. (2)].

Over the last two decades, it has become clear that antibodies against neuronal surface antigens, particularly receptor-gated ion channels of ion-channel-associated proteins, can reach the brain to cause a group of disorders referred to as antibody-mediated or autoimmune encephalitis (AE) (1). These are immune disorders of the central nervous system (CNS) characterized by a wide range of neurological and psychiatric clinical features and associated with antibodies against different proteins expressed on the neuronal surface, mainly at excitatory, and inhibitory synapses (Figure 1). Distinct from classical paraneoplastic syndromes that are associated with onconeural antibodies (3), in AE, the neuronal surface antibodies (NSAbs) are considered to be pathogenic, and patients respond substantially to immunotherapies that reduce antibody levels (4).

**Figure 2:**
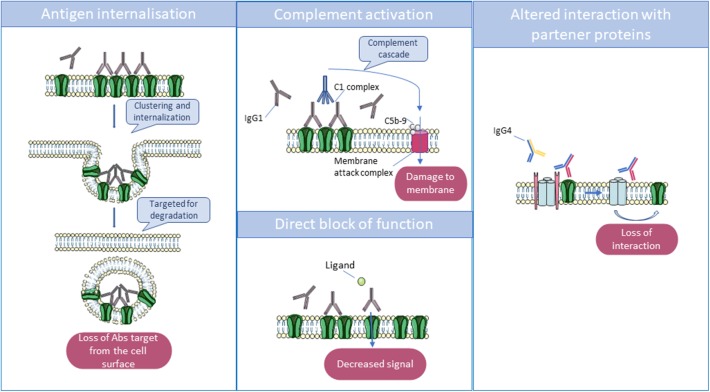
Main mechanisms by which antibodies act to reduce the function of their targets. Immunoglobulin G1 (IgG1) and IgG3 can cross-link antigenic targets, leading to internalization, and degradation of the antigen in lysosomes. Also, IgG1 and IgG3 can activate the complement cascade via their Fc domains, which interact with complement proteins C1 and C1q. The complement cascade culminates in the formation of the membrane attack complex which disrupts the phospholipid bilayer, resulting in cell damage. Finally, some autoantibodies can directly block receptors by binding to an essential transmitter or regulatory binding site, but monovalent IgG4 can only act by disrupting the function of the target or the interaction between their target and partner proteins.

Interestingly, these pathogenic antibodies can be either predominantly immunoglobulin G1 (IgG1) or IgG4, depending on the target antigen. *In vitro* studies have helped to decipher the mechanisms by which they lead to neuronal dysfunction: in many cases, divalent antibodies (IgG1 > IgG3, IgG2) cause internalization of adjacent surface proteins, leading to their loss from the membrane; complement activation by these antibodies can be demonstrated *in vitro* but may not always occur *in vivo*. By contrast, in some disorders, IgG4 antibodies predominate and act principally or exclusively by direct inhibition of the function of the target antigen [see (5) and Figure 2].

However, an effect of the antibodies *in vitro* does not necessarily reflect a pathogenic role *in vivo*. For instance, IgG, IgA, and IgM *N*-methyl-d-aspartate receptor (NMDAR) antibodies (NMDAR-Abs) have been identified in a small proportion of healthy humans and mammals (6–9) and cause internalization of the NMDAR in cultured neurons (9, 10), similar to the antibodies found in patients with the IgG NMDAR-Ab encephalitis (NMDARE) (11). This suggests that other factors are likely required to induce the clinical syndrome, factors that may be difficult to model *in vitro* alone.

Indeed, according to the modified Witebsky criteria (12), direct and indirect evidence of pathogenicity requires the reproduction of the disease in a recipient through direct transfer of the antibodies (passive transfer) or through active immunization, respectively. Animal models not only provide evidence of pathogenicity but can also offer insight into sites of action, pathogenic mechanisms, and therapeutic approaches.

Accordingly, over the last few years, animal models, usually in mice, have been established for the most commonly encountered NSAbs in clinical practice. Below, we describe the approaches used and the results of these models and discuss their advantages and limitations. We also summarize recent experiments that demonstrate that maternal antibodies to these or other NSAbs can alter development in the offspring with potential lifelong susceptibility to neurological or psychiatric diseases.

## Different Models of Antibody-Mediated Disorders

Animal models of autoimmune disorders can be divided into two main categories: (1) spontaneous models where, comparably to humans, animals develop an autoimmune disease spontaneously and (2) induced models where an autoimmune disease is artificially provoked. Spontaneous forms of AE have been reported in different species, but they are uncommon (13, 14). Most of the models of AE have been obtained through induction by passive or active immunization. Passive immunization is based on the reproduction of the disease in a healthy recipient by transfer of serum, purified immunoglobulins, monoclonal antibodies, or, more rarely, antibody-producing cells isolated from an affected human or animal donor. Active immunization is based on the exposure to an antigen, often in association with adjuvants, to generate an adaptive immune response. The antigen can be in the form of purified proteins, recombinant or synthesized peptides (15).

Work on myasthenia gravis (MG) provides examples of both active and passive immunization and has helped to shape our understanding of antibody-mediated diseases (Table 1). Passive transfer is the best way to assess the acute effects of human autoantibody-mediated diseases and has been used extensively to study patients' derived antibodies in MG [(16); see a brief review by Phillips and Vincent (17). By contrast, active immunization (see (18)] has been particularly useful to investigate more broadly the immunological factors underlying the disease, though with the limitations of possible differences between the function of the human and rodent immune systems and between different strains of mice. For instance, C57B1/6 mice were very susceptible to active immunization with acetylcholine receptor (AChR), whereas AKR/J mice were resistant (19, 20). Moreover, the use of the target antigen as a whole protein often induces high titers of antibodies, but if the protein is from a different species, not all of the antibodies will necessarily cross-react with the mouse antigen or be directed against the disease-causing epitope(s). Therefore, active immunization models are not always relevant to the human pathology but, when successful in producing an appropriate clinical and physiological phenotype, provide a long-term model of the disease that is suitable for testing experimental therapies.

**Table 1:** Example of antibody-mediated diseases: clinical and experimental evidence for MG.

**IN HUMANS:**
Clinical features (weakness and fatigue) can be reversed by plasma exchange and other immunotherapies (21). IgG1 and IgG3 antibodies to the AChR are present in the majority of patients (22, 23). IgG and complement deposition are found at the neuromuscular junction (24). The thymus gland contains germinal centers and produces some of the AChR antibodies (25). Thymectomy leads to long-term clinical benefit, reducing the need for immunotherapies (26). Mothers can transfer pathogenic antibodies to the fetus or neonate, causing a transient form of MG (27) or rarely a severe neurodevelopmental disorder (arthrogryposis multiplex congenital) (28).
**GENETIC CONDITIONS:**
Genetic conditions caused by mutations in genes encoding AChRs cause similar clinical features but without evidence of autoimmunity. Genetic conditions can be modeled in transgenic mice [see (29)].
**IN EXPERIMENTAL ANIMALS:**
Injection of patient IgG into mice or other species leads to short-term clinical or electrophysiological evidence of the disease (16). Active immunization against purified AChRs leads to a more severe and prolonged model (30).

*For a brief review of the history of research into myasthenia gravis, see Vincent (31). MG, myasthenia gravis; IgG, immunoglobulin G; AChR, acetylcholine receptor*.

In contrast to conditions such as MG, where the target antigens of the antibodies are peripheral and thereby easily accessible from the systemic circulation, the blood–brain barrier (BBB) limits the access of immune molecules to the brain. One way to overcome this limitation, in models of CNS antibody-mediated diseases, is to infuse the antibodies directly into the cerebrospinal fluid (CSF) within the cerebral ventricle(s) (intracerebroventricular, icv) or to inject them into the brain parenchyma. However, in the majority of autoimmune forms of encephalitis, the antibody levels are higher in the serum than in the CSF, suggesting that the antibodies could initiate the disease by diffusion through an incomplete or temporarily disrupted BBB (32) or at sites of limited BBB protection such as the choroid plexus. Therefore, another approach is to administer the antibodies in the periphery, using the intravenous (iv) or intraperitoneal (ip) route and if necessary to induce artificially a breach in the BBB to allow the antibodies to reach their targets. Classically, the latter is obtained by one or two ip injections of lipopolysaccharide (LPS), which induces a transient disruption of the BBB, particularly in the frontal cortex, thalamus, pons–medulla, and cerebellum (33). It is not yet clear whether the icv or ip route of administration is most appropriate and whether they could lead to different CNS changes.

Finally, there is a possibility of transfer from a mouse dam to developing embryos. Although the BBB interfaces are formed early in development (34), maternal IgG antibodies can cross into the fetal brain parenchyma during gestation (32). It is long established that a neonatal form of MG can result from the transfer of IgG antibodies from an affected mother to her fetus *in utero* (27, 35). Human MG AChR antibodies injected intraperitoneally into pregnant mice were shown to cross efficiently from the mouse dam to her fetuses and to cause neuromuscular changes *in utero* (36); this model has since been used to study the effects of human serum antibodies on brain development (as described below).

## Models of Neuronal Antibody-Mediated Disorders

The clinical and investigative features of the patients with antibodies to neuronal surface proteins, and the results of the existing models, are summarized in Table 2.

**Table 2:** Summary of main features of NSAb diseases and the models.

**Clinical features**	**Investigations**	**Main mechanisms identified *in vitro***	**Active or PT**	**Animals, route, duration**	**Material**	**Behavior and other observations**	**Pathology**	***Ex vivo* physiological studies**	**References to *in vitro* and *in vivo* models**
**NMDAR (IgG1 PREDOMINANTLY)**									
NMDAR encephalitis: psychiatric syndrome, seizures, amnesia, movement disorders catatonia, autonomic instability	EEG variable MRI often normal CSF cellular, intrathecal synthesis		Active	C57BL/6 mice (12 months old) WT and ApoE^−/−^; single injection of a mixture of GluN1 extracellular peptides and/or chicken ovalbumin + complete Freund's adjuvant	NMDAR1 peptides	Hyperactivity only after MK-801 in APOE^−/−^ mice 4 weeks after immunization	No CD3 infiltrates, no microglia activation	NA	(9)
		After 24-h incubation with serum from proteoliposome-treated mice, cultured hippocampal neurons showed reduced NMDAR-mediated currents and a decrease of >50% in GluN1 immunoreactivity	Active	C57BL/6 adult mice; subcutaneous injection of NMDARs in proteoliposomes (or liposomes or saline) followed by a booster 2 weeks later	Purified GluN1/GluN2B NMDA fully assembled tetrameric receptors (holoreceptors) embedded in liposomes	Hyperactivity, stereotypied, and anxiety-like behavior 4 weeks after immunization; overt seizures (21%), and hunched back/lethargy (11%)	Perivascular cuffing; patchy areas of cell death; microgliosis; immune cell infiltrates in the brain	Reduced NMDAR-mediated currents in cultured hippocampal neurons incubated with serum of immunized mice	(37)
		Internalization of NMDARs Loss of NMDARs Disruption of ephrin interaction	PT	Male C57BL/6J mice (8–10 weeks old); icv infusion over 14 days	Pooled CSF	Cognitive and depressive-like	IgG bound, NMDAR loss	NA	(38) (11)
			PT	icv, single bolus	Purified serum IgG	Increased seizure susceptibility	IgG, no NMDAR loss	Seizures after PTZ	(39)
			PT	Male C57BL/6 mice (age 8 weeks); icv infusion over 18 days	CSF from patients with NMDARE	Impaired spatial memory as detected with the Morris water maze test	Decreased content of NMDAR in the hippocampus; no neuronal loss or inflammatory cell infiltrates; increased CXCL10 expression in the brain	NA	(40)
			PT	Male C57BL/6J mice (8–10 weeks old); icv infusion over 14 days	CSF from patients with NMDARE with or without ephrin-B2	Memory deficit and depressive-like behavior. EphB2 prevented antibody effects	Decrease of the density of cell surface and synaptic NMDAR and EphB2	Impairment of long-term synaptic plasticity	(41)
			PT	Male C57BL/6 mice (8–10 w old); ICV infusion over 14 days	CSF or IgGs purified from CSF of patients with NMDARE	Absence of overt changes in memory (NOR), anxiety, and locomotor activity (OF, RT). However, reduced preference for novel object at NOR	No neuronal loss; astrocytic hypertrophy but not proliferation in the hippocampus	Increased frequency of seizures; reduced excitability and membrane resistance of CA1 pyramidal neurons in mice hippocampal slices	(42)
		Patient-derived rhuMAb, specifically synaptic NMDAR clusters in cultured hippocampal neurons and NMDAR-mediated currents in NMDAR transfected cells	PT	Mice; icv infusion over 14 days	Recombinant human antibodies from clonally expanded intrathecal plasma cells	Memory impairment at NOR test	Human IgG bound; NMDAR loss in the hippocampus	NA	(43)
		mAb caused internalization of NMDAR	PT	Female Swiss Webster mice, 6–8 weeks old; single iv injection + LPS; 4 days' observation after 3 days' recovery	mAb from a patient with NMDARE	increased spontaneous locomotor activity	NA	NA	(44)
			PT	Female BALB/c mice (8–10 weeks old)	Intranasal inoculation of HSV-1 + ACV	NA	4/6 mice developed serum NMDAR-Abs and showed decreased brain NMDAR expression	NA	(45)
			PT	Males Wistar rats; single stereotactic injection in the hippocampus (CA1) and premotor cortex	CSF or IgGs purified from CSF of patients with NMDARE	Increased glutamate	NA	NA	(46)
			PT	Female Wistar rats (2 months old); single stereotactic injection in the hippocampus (dentate gyrus)	CSF of patients with NMDARE or commercial anti-NMDAR1-Ab	Impaired memory at Morris water maze	NA	Reduced LTP in the dentate gyrus; absence of increased frequency of recurrent epileptiform discharges induced by gabazine compared with controls	(47)
			PT	Female Wistar rats (60–90 days old); single stereotactic injection in the hippocampus (CA3)	CSF of patients with NMDARE	NA	NA	Reduced LTP magnitude at A/C fiber-CA3 synapses compared with controls; increased frequency of epileptiform after potentials following the fEPSP	(48)
			PT	Female Wistar rats (8–10 weeks old); single stereotactic injection in the hippocampus (CA1)	CSF of patients with NMDARE	Absence of overt alteration at NOR, locomotor activity, and anxiety. However, reduced preference for NO at NOR	NA	Schaffer collateral–CA1 LTP reduced in hippocampal slices	(49)
**CASPR2 IGg4 > IGg1**									
Peripheral (pain, neuromyotonia, autonomic dysfunction)	EMG evidence of peripheral nerve hyperexcitability	Loss of Kv1 expression on the surface of cultured DRG neurons incubated with CASPR2-IgG	PT	Male C57BL/6J mice (8–10 weeks old); ip daily injections for 14–18 days	Purified plasmapheresis IgG	Evidence of lowered thresholds for mechanical pain	IgG bound in DRG, small increase of microglia in spinal cord	Decreased Kv currents with increased excitability of DRG neurons	(50)
Central: limbic encephalitis, Morvan's syndrome	MRI FLAIR hippocampal hyperintensity, CSF bland, little intrathecal synthesis	Some internalization of CASPR2 but no loss of surface CASPR2	PT	Male C57BL/6J mice (8–10 weeks old); ip daily injections for 8 days + 1 ip LPS injection	Purified plasmapheresis IgG	Modest loss of working memory, abnormal behaviors in the presence of novel mouse	No loss of CASPR2 but extensive microglial activation and astrocyte activation with complement expression	NA	(51)
		CASPR2 internalization with reduction of CASPR2 surface expression and decreased intensity of surface GluA1 total and synaptic clusters	PT	C57BL/6J mice; single stereotactic injection; primary visual cortex (V1)	Purified IgG from PLEX	NA	NA	Reduced amplitude of AMPAR-mediated mEPSCs in V1-layer 2/3 pyramidal neurons incubated with patient IgG	(52)
**LGI1 IGg4 > IGg1**									
Central: LE with or without FBDS and or hyponatremia	MRI FLAIR hippocampal hyperintensity, usually normal CSF, rare OBs; Abs can be absent	Antibodies prevent the binding of LGI1 with ADAM22 and ADAM23	PT	Male C57BL/6J mice (8–10 weeks old); icv infusion over 14 days	Purified IgG from serum	IgG bound; reduced Kv1.1 and AMPAR	Memory deficit at NOR	Increased presynaptic excitability and glutamatergic synaptic transmission and impaired LTP in acute hippocampal slices from LGI1-IgG-injected mice	(53)
**AMPAR**									
Central: LE	Lymphocytosis; OBs; Abs usually present	Internalization of AMPARs; depletion of heteromeric synaptic AMPARs containing GluA2 most likely followed by a synaptic incorporation of GluA1 homomeric AMPARs; decreased mEPSC amplitudes and frequency in neurons treated with a-GluA2 IgG	PT	C57BL/6 mice (WT and GluA1-KO); icv infusion over 14 days or single stereotactic intrahippocampal (CA1) injection	IgG purified from serum	Memory impairment at NOR and anxiety-like behavior (maximum effect after 18 days during pump infusion)	IgG bound to hippocampus; unchanged spine density and morphology; downregulation of GluA2	Reduced mEPSC amplitudes and impairment of LTP in the SC- CA1 pathway in acute hippocampal slides	(54)
**GlyR MAINLY IgG1**									
Brain stem and spinal cord: PERM or SPS	Often no evident MRI or EEG findings. Pleocytosis in half of the cases, OBs (20%)	Cause internalization of GlyRs in transfected HEK cells. Inhibit GlyR function acutely	PT	ip daily injections of >10 mg/day for 11 days with 2 LPS injections	Purified plasmapheresis IgG	Modest motor phenotype with poor performance on rotarod and on narrow rods	IgG bound to brain stem and ventral horns IgG detected inside large brain stem neurons GlyRs persisted on surface of neurons	NA	(55) (Carvajal-Gonzalez et al., unpublished data)

## NMDAR-Ab Encephalitis

### Clinical Disease and *in vitro* Mechanisms

NMDARE, the classical syndrome associated with IgG1 NMDAR-Abs, is the most commonly recognized AE in clinical practice. It is characterized by psychiatric symptoms, such as confusion, abnormal behavior, paranoia, and hallucinations, in addition to memory problems, seizures, dyskinesia, autonomic instability, catatonia, hypoventilation, lethargy, and language deficits (56). *In vitro*, pathogenic NSAbs bind and cause clustering (57), cross-linking, and internalization of NMDAR, leading to a loss of functional receptors on the cell surface (NMDAR hypofunction), which is reversible on removal of the NMDAR-Abs (11). Moreover, NMDAR-Abs induce dispersal of GluN2A-NMDAR, through the blockade of the interaction between the extracellular domains of GluN1/GluN2 subunits and ephrin-B2 receptors (EPHB2R) (58).

In a high proportion of younger women, the disease is caused by the presence in an ovarian teratoma of neuronal tissue expressing NMDARs and inducing an immune response (59, 60). In others, particularly young children, the disease can follow herpes simplex virus encephalitis (HSVE), probably as a secondary response to the neuronal damage caused by the virus (61).

### Spontaneous or Genetic Disease

NMDAR-Abs have been described in other mammals (9) and are present at a low percentage (around 1%) in healthy individuals. In 2014, a retrospective study showed that Knut, the polar bear of the Berlin Zoological Garden who drowned in 2011 following seizures, had high levels of NMDAR-Abs in his serum and CSF, making him the first non-human case of NMDARE and reaffirming the epileptogenicity of these antibodies in mammals. Pathological examination showed a patchy distribution of infiltrating immune cells, with numerous plasma cells around vessels and within the parenchymal infiltrates, in the absence of marked neuronal abnormalities (14).

Mutations in GRIN1 [which encodes the GluN1 (NR1) subunit of NMDAR] have been associated with a phenotype consisting of severe intellectual disability, seizures, hyperkinetic and stereotyped movement disorders, and dysmorphic features (62–64). In mice, selective deletion of GluN1 in CA1 and CA3 pyramidal neurons abolished long-term potentiation (LTP) and induced memory impairment (65, 66).

### Passive Transfer Models

Animal models of NMDARE have been published recently with results that recapitulate some of the specific features of the human disease. In rats, stereotactic parenchymal injection of CSF or purified IgGs from patients with NMDARE produced different outcomes. Infusion in the CA1 and premotor cortex increased the levels of extracellular glutamate and, consequently, neuronal excitability (46). On the other hand, several studies showed that a single injection of CSF from patients with NMDARE into the hippocampus produced a reduction of LTP in the CA1, CA3, and dentate gyrus (47–49). Behaviorally, effects ranging from impaired Morris water maze memory performance (47) to a lack of novel object recognition (49) were reported, in the absence of significant changes in locomotor activity or anxiety-like behavior (49).

Continuous icv infusions of CSFs pooled from individuals with NMDARE into mice over 14 days reproduced some of the neuropsychiatric features observed in patients such as memory deficits, anhedonia, and depressive-like behaviors. Seizures or movement disorders were not observed. IgG deposition and a decrease in NMDAR clusters on hippocampal neurons was observed in NMDAR-Ab-injected mice, which resolved within days after discontinuing the infusion (38). Further studies have also shown disruption of the normal interaction with other synaptic proteins, in particular EphrinB2R. Administration of ephrin-B2 (the ligand of the EphrinB2 receptor) in the 14-day infusion animal model prevented the pathogenic effects of NMDAR-Abs on memory and behavior, levels of cell-surface NMDAR, and synaptic plasticity (41). Recently developed human-derived monoclonal antibodies to the NMDAR have produced similar pathogenic effects *in vivo* and *in vitro* and offer a promising less-limited resource (compared to human CSF and IgG) for future experimental studies (43).

**Figure 3:**
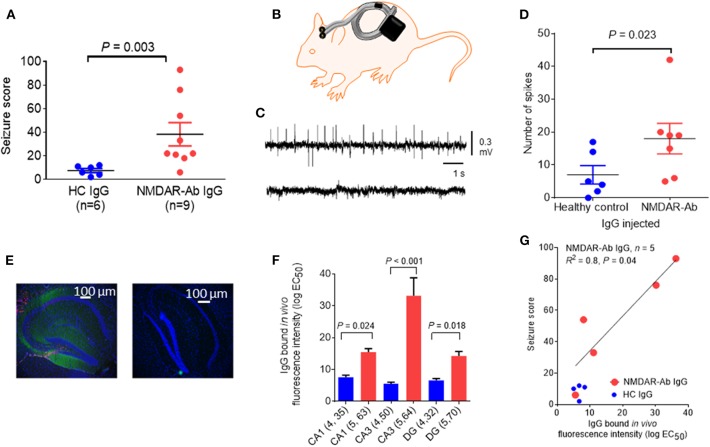
Epileptogenic effects of a single intracerebroventricular (icv) injection of *N*-methyl-d-aspartate receptor antibody (NMDAR-Ab)-positive immunoglobulin G (IgG). **(A)** The seizure score of mice injected with NMDAR-Ab IgG was higher than that of those injected with control IgG following exposure to a subthreshold dose of PTZ. **(B)** Diagram showing placement of subcutaneous wireless electroencephalogram (EEG) transmitter that allows continuous EEG recording in injected mice with no need for tethering (Open Source, Hashemi Instruments, USA). **(C)** A representative EEG of an NMDAR-Ab-injected mouse post-PTZ shows a number of “spikes” corresponding to convulsive seizures (upper trace), compared with the EEG of a healthy control IgG-injected mouse, which has minimal spike activity (lower trace). **(D)** When analyzed using the computer-based event detection program and blinded observer verification, the number of spikes seen in the hour following PTZ injection was greater in the NMDAR-Ab (*n* = 7) compared with the healthy control IgG (*n* = 6) injected mice (*P* = 0.023, Mann–Whitney). Results are mean ± SEM. **(E)** Human IgG injected *in vivo* was detected postmortem in NMDAR-Ab IgG-injected mice with antihuman IgG (green) merged with the nuclear stain 4′,6-diamidino-2-phenylindole (DAPI) (blue). The typical pattern of NMDAR-Ab in the molecular cell layer with sparing of the granule cell layer was found (left image). Control IgG-injected mice had no detectable IgG (right image). **(F)** Bound human IgG in the hippocampi, as determined by the mean fluorescence intensity analysis of brain sections, was higher in the NMDAR-Ab IgG-injected mice than in healthy control IgG-injected mice in the CA1, CA3, and dentate gyrus (DG). **(G)** For the NMDAR-Ab animals (*n* = 5), there was a linear correlation between IgG binding and seizure score (*R*^2^ = 0.8; *P* = 0.04). The contents of this figure are taken from Wright et al. (39) with permission from Oxford University Press.

In another mouse model, icv injection of purified plasmapheresis IgG from individuals with NMDARE induced, in association with a subthreshold dose of the chemo-convulsant pentylenetetrazol (PTZ), more frequent and severe seizures than a single injection of IgG from control individuals [(39); see Figure 3]; cognitive and other features were not examined in these mice. Continuous wireless electroencephalogram (EEG) recording did not identify any spontaneous seizure activity. However, there was IgG bound to the hippocampus at 48 h post icv infusion, particularly to the CA3 region, and it correlated with the number and severity of seizures seen in the mice, but there was no apparent loss of NMDARs (Figure 3). In a more recent study, EEG recordings of mice infused intraventricularly for 14 days with CSF NMDAR-Abs showed a higher frequency of seizures compared with control mice, associated with variable behavior ranging from sleeping or normal exploratory activity to freezing and myoclonic jerks (42). Two main seizure patterns were observed, one, more frequent, characterized by high-amplitude rhythmic spikes that occurred at relatively constant rates or at irregular intervals and another, less common, characterized by high-amplitude fast rhythmic activity that fluctuated in amplitude in a spindle-like fashion (42). Continuous EEG recordings may be necessary to detect reliably spontaneous non-motor seizures in models of antibody-mediated encephalitis. Neuropathology showed absence of neuronal death and only mild astrocytic activation (42).

In another study using continuous icv infusion, mice receiving patients' CSF showed memory impairment in the Morris water maze, but not in the novel object recognition test, and a tendency to a reduced expression of NMDAR in the mouse brains. No overt inflammatory changes were observed, but an increase of the chemokine CXCL10 was detected (40), a finding that has been observed also in patients with NMDARE (67). Intravenous infusion of monoclonal NMDAR-Abs followed by LPS increased mouse voluntary locomotor activity at the mouse wheel-running test, similarly to that observed in mice treated with low doses of the NMDAR inhibitor MK-801 (44).

Overall, the passive-transfer animal models support the proposed mechanisms of cross-linking and internalization as well as the relevant role of altered NMDAR trafficking in the pathogenesis. However, these models have not demonstrated all the clinical features; for example, none have reproduced the (often-striking) movement disorders or shown long-term cognitive deficits and structural hippocampal damage as seen in some patients (68). A possibility is that some inflammatory changes are not reproduced by passive transfer. The discrepancies observed between different models might also relate to different protocols, to the use of different species and strains, and to different effects of the antibodies in relation to acute or chronic exposure.

### Active Immunization

In a recent mice active immunization model, Pan et al. (9) showed that mice immunized against NMDAR1 peptides did not show behavioral changes at the open-field test. Even in the presence of high titers of NMDAR-Ab, an increase of locomotor activity, a psychosis-like behavior, was obtained only upon MK-801 challenge in ApoE^−/−^ mice, which present a disrupted BBB. No lymphocyte (CD3) infiltrates nor microglial activation was detected on immunopathology. On the contrary, immunization with purified GluN1/GluN2B fully assembled tetrameric NMDARs (holoreceptors) embedded in liposomes induced a phenotype characterized by hyperactivity, stereotyped motor features (tight curling), and seizures in association with neuroinflammation and immune cell infiltrates (37). Distinct from the passive-transfer models, these immunized mice produced GluN1 and GluN2 antibodies that reacted with the linear epitopes of the NMDAR protein, and not the amino-terminal domain of GluN1 as seen in the human-derived antibodies (69). Nevertheless, this model may prove useful for testing novel treatments acting on the cellular inflammatory component of the disease.

Finally, a recent small study investigated the mechanisms involved in the pathogenesis of post-HSV-1 NMDARE (45). Following intranasal inoculation of HSV-1, 67% (four out of six) of mice developed serum NMDAR-Abs. The same mice showed reduced hippocampal NMDAR compared with mice without antibodies, inferring IgG-mediated loss, but the authors did not demonstrate IgG antibodies bound to the hippocampus. This model could be a useful platform to further explore the mechanisms of post-HSV encephalitis with secondary NMDARE.

## CASPR2-Ab Encephalitis

### Clinical Disease and *in vitro* Mechanisms

CASPR2 is a neurexin-related cell adhesion molecule expressed in the CNS and peripheral nervous system, and CASPR2 antibodies (CASPR2-Abs) react with both the brain and peripheral nerve tissues [(70); see Figure 1]. This expression pattern well-explains why CASPR2-Abs have been associated not only with peripheral nerve hyperexcitability (often called neuromyotonia) but also with CNS symptoms including cognitive impairment, memory loss, hallucinations, delusions, cerebellar symptoms, and epilepsy. Some patients present with Morvan syndrome (MoS), characterized by the combination of neuromyotonia, neuropathic pain, encephalopathy with hallucinations, and a sleep disorder, described as agrypnia excitata (71, 72); the latter is characterized by severe insomnia, dream-like stupor (hallucinations and enacted dreams), sympathetic hyperactivity (hyperthermia, perspiration, tachypnea, tachycardia, and hypertension), and motor agitation. CASPR2-Abs are mainly IgG4, but most patients have IgG1 antibodies as well.

CASPR2 is essential for clustering Kv1.1 and Kv1.2 channels at the juxtaparanodes of myelinated axons, where the channels are important for repolarization of the nerve axon, avoiding repetitive firing and helping to maintain the internodal resting potential. Their functions at CNS synapses are not well-defined.

The *in vitro* effects of CASPR2-Abs are complex. In one study, the antibodies inhibited CASPR2 interaction with contactin-2 but did not lead to CASPR2 internalization (73). However, in two others, *in vitro* exposure induced CASPR2 internalization *in vitro* (51, 52) with variable effects on CASPR2 expression, ranging from absent (51) to significant (52) loss of surface expression.

### Spontaneous or Genetic Disorders

Interestingly, mutations in the *CNTNAP2* gene, encoding CASPR2, are associated with focal epilepsy, schizophrenia, and autism spectrum disorder (ASD) (74). CNTNAP2-knockout (KO) mice were shown to have social deficits, abnormal motor activity, cognitive deficits, and seizures (75).

### Passive Transfer Models

Intraperitoneal injection of purified IgG from two CASPR2-Ab-positive patients to mice over 14–18 days, without attempt to breach the BBB, reduced the thresholds for mechanical stimuli, a signature of pain (50). The effects induced by the antibodies on pain sensitivity were also observed in KO mice lacking CASPR2 (CNTNAP2^−/−^). These mice demonstrated enhanced pain-related hypersensitivity to noxious mechanical stimuli, although more severe than that obtained with the antibodies, and also to heat and algogens. Nevertheless, either immune or genetic-mediated ablation of CASPR2 enhanced the excitability of dorsal root ganglia (DRG) neurons through regulation of Kv1 channel expression at the soma membrane (50). CASPR2-IgG did not cause neuronal loss nor overt inflammation, although a modest increase in microglial cell count was observed in the spinal cord (50).

**Figure 4:**
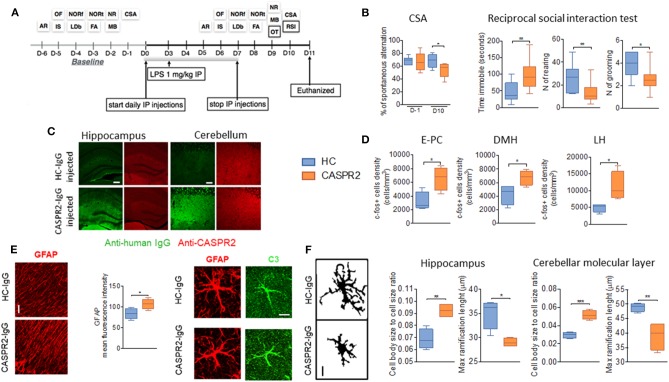
Intraperitoneal (ip) injection of CASPR2 immunoglobulin G (IgG) with lipopolysaccharide (LPS) causes behavioral and neuropathological changes in mice. **(A)** Experimental design and selected behavioral tests. The behavioral tasks assessed locomotion (open field, OF), strength (inverted screen, IS), coordination (accelerating rotarod, AR; and narrow beam, NB), working memory (continuous spontaneous alternation, CSA), short- (forced alternation, FA) and long-term memory (novel object recognition, NOR—NORf, familiarization phase; NORt, test phase), anxiety (light-dark box, LDb), compulsive-like behavior (marble burying test, MB), social behavior (reciprocal social interaction tests, RSI), and olfaction (olfaction test, OT). **(B)** Continuous spontaneous alternations were reduced in CASPR2-IgG-injected mice compared with HC-IgG-injected mice (*P* = 0.044). In the RSI test, there was reduced latency to interact (*P* = 0.04; Mann–Whitney test) but no differences in the interaction time or number of interactions. However, in the non-social aspects of the test, there was increased time spent immobile (*U* = 0.008), reduced rearing (*U* = 0.02), and reduced grooming (*U* = 0.018). **(C)** Bound human IgG in the hippocampi and cerebellum of CASPR2- and HC-IgG-injected mice. CASPR2-IgG-injected animals had higher levels of IgG in the cortex (Cx) (*P* = 0.03), hippocampus (Hip) (*P* = 0.023), and thalamus (Th) (*P* = 0.0004) compared with HC-IgG-injected mice. No differences were observed in the levels of CASPR2 expression in the same areas (*n* = 4 per group). **(D)** c-Fos expression in the entorhinal–piriform cortex (*P* = 0.020), dorsomedial hypothalamus (DMH) (*P* = 0.037), and lateral hypothalamus (LH) (*P* = 0.031) was higher in the CASPR2-IgG-injected mice than in the HC-IgG-injected mice (*n* = 4 per group). **(E)** Representative images of glial fibrillary acidic protein (GFAP) staining in the molecular layer of the cerebellum and quantification of the mean fluorescence intensity in the same area showing higher GFAP expression in the CASPR2-IgG-injected mice (*P* = 0.043) (*n* = 4 per group; 40X, 10 μm). On the right, representative images of complement C3 expression on GFAP-positive cells. Percentage of C3/GFAP area ratio per cell showed increased C3 expression of astrocytes in CASPR2-IgG-injected mice. **(F)** Representative images of the z-stack projected IBA1 staining used for morphological analysis (40X, 10 μm). Quantification of morphological data in the hippocampus and molecular layer of the cerebellum showed that microglia from CASPR2-IgG-injected mice had a higher cell soma/cell total body size ratio [*t*_(6)_ = 4.74, *P* = 0.0032] and shorter [*t*_(6)_ = 3.68] ramifications than HC-IgG-injected mice, compatible with an activated phenotype in both the hippocampus (*P* = 0.017 and *P* = 0.010, respectively) and the cerebellum (*P* = 0.0003 and *P* = 0.008, respectively). ^***^The contents of this figure are taken from Giannoccaro et al. (51) with permission from Oxford University Press. ^*^ < 0.05, ^**^ ≤ 0.01, ^***^ ≤ 0.001.

To explore the effects of CASPR2-Ab in the CNS, a similar protocol was used with eight daily injections of IgG purified from one patient with AE and from one healthy control (Figure 4). A single dose of LPS was added at day 3 to disrupt the BBB (51). Mice injected with CASPR2-IgG showed less alternation in the continuous spontaneous alternation tests, suggestive of memory impairment, and longer latency to interact and increased immobility during the social interaction test (Figure 4). These changes had not been seen during isolated open-field or other tests, suggesting that the effects could be indicative of anxiety in the context of a novel mouse, rather than an effect on normal exploratory activity. At neuropathology, CASPR2-IgG injected mice showed human IgG deposition, particularly in the cortex, hippocampus, and thalamus; mild loss of Purkinje cells and c-Fos activation as well as microglial and astrocyte activation without B- or T-cell infiltration (Figure 4). Microglial activation has been reported in neuropathological cases of patients with CASPR2-Ab encephalitis (76, 77).

Although this model showed evidence of pathogenicity of CASPR2-IgG, it failed to recapitulate the wide range of defects found in the patients (e.g., autonomic, sleep disturbance, and hormonal/neuropeptide abnormalities) who would require substantial additional tests. Moreover, it does not explain how CASPR2-Abs cause their effects. Indeed, IgG deposition was not associated with a reduction of CASPR2 expression on immunohistology. On the contrary, a trend toward higher levels of mouse CASPR2 was seen in the brain extracts of CASPR2-IgG-injected mice, suggesting some compensatory upregulation.

Injection of a mixture of CASPR2-Abs in mouse visual cortex produced impaired localization of mouse Caspr2 to excitatory synapses and significantly decreased AMPAR-mediated currents in layer 2/3 pyramidal neurons; this implied a dysfunction of glutamatergic transmission in the pathogenesis of CASPR2-Ab encephalitis (52). Future studies should evaluate in parallel the effects of CASPR2-Abs on its partner protein network and on neuronal activity.

## LGI1-Ab Encephalitis

### Clinical Disease and *in vitro* Mechanisms

Autoantibodies to LGI1 (LGI1-Abs) are the most common autoantibody in patients with limbic encephalitis (LE), a clinical syndrome characterized by the acute development of mood changes, anxiety, short-term memory deficit, and seizures due to an inflammatory process involving the limbic system that includes the medial temporal lobes, hippocampus, amygdala, and frontobasal and cingulate cortices (1). In patients with LGI1-Abs, the onset of an overt limbic dysfunction can be preceded by episodes of faciobrachial or crural seizures that last a few seconds and occur many times during the day; these episodes have been described as faciobrachial dystonic seizures (FBDS) (78).

LGI1 is a protein secreted by the presynaptic terminals of neurons that bind to ADAM22 and ADAM23, two proteins involved in cell–cell adhesion and located presynaptically and postsynaptically, respectively (Figure 1). Binding to ADAM22, LGI1 regulates AMPAR-mediated synaptic currents in the hippocampus (79). Binding to ADAM23, LGI1 selectively prevents inactivation of the presynaptic voltage-gated potassium channel Kv1.1 (80) mediated by a cytoplasmic regulatory protein, Kvβ (81).

In cultured hippocampal neurons, LGI1-Abs disrupt the ligand–receptor interaction of LGI1 with ADAM22, resulting in reversible reduction in synaptic AMPARs [(82); see Figure 1]; these antibodies could be IgG4. However, in the few postmortem studies on patients who have died unexpectedly, there appears to be IgG deposition, some complement deposition, and loss of neurons. These findings would be compatible with the presence of IgG1 antibodies; although they are in the minority compared with IgG4, they tend to be much higher in patients with cognitive impairment (70, 83). IgG1-induced neuronal loss would explain why, despite a good response to immunotherapy, many patients are left with hippocampal atrophy (84), and only 35% of patients return to their baseline cognitive function (85).

### Spontaneous or Genetic Disorders

LGI1 mutations have been associated with an autosomal dominant lateral temporal lobe epilepsy (ADLTLE) manifesting with focal seizures often with auditory features (86). The majority of mutations prevent LGI1 secretion, whereas others alter its interactions with ADAM22/ADAM23 (87). Animal models of LGI1 depletion all present spontaneous seizures (88–92), although the mechanisms behind this increased epileptic susceptibility have not been fully elucidated and both enhanced excitatory transmission (90, 91, 93) and reduced AMPAR function (79, 80, 89) have been reported.

Intriguingly, a spontaneous model of LGI1-Ab encephalitis has been observed in cats with feline complex partial seizures with orofacial involvement (FEPSO) (13, 94–96). Clinically, they presented with acute onset of complex partial seizures with orofacial involvement (salivation, facial twitching, lip smacking, chewing, licking, or swallowing), motor arrest (motionless starring), and behavioral changes associated with bilateral hyperintensities at brain MRI (13, 94, 95). Postmortem analysis of three cases showed IgG and complement deposition associated with neuronal loss, consistent with the findings in the few available postmortem examinations from patients with LGI1-related encephalitis (3, 95). Subsequent neuropathological studies in cats showed also that, whereas T-cell infiltrates were present brainwide, BBB leakage was more restricted to limbic areas (96). This observation suggests that a local BBB vulnerability might be responsible for the selective involvement of the limbic system, even though LGI1 is expressed throughout the brain.

### Passive Transfer Model

More recently, the pathogenicity of LGI1-Abs has been confirmed by a passive transfer mouse model based on cerebroventricular transfer of patient- or control-derived IgG (53). LGI1-Ab-injected mice showed memory impairment which slowly reversed after stopping the infusion. However, in contrast to the spontaneous feline model and LGI1-KO animals, no epileptic seizures were observed. Nevertheless, LGI1-Ab caused a significant decrease of the density of total and synaptic Kv1.1 and AMPAR clusters due to the disruption of LGI1 interactions with presynaptic ADAM23 and postsynaptic ADAM22. Consistent with decreased Kv1.1 expression and previous *in vitro* studies (97), increased presynaptic excitability and glutamatergic transmission were observed in acute brain slice preparations, resulting in increased evoked excitatory postsynaptic currents (eEPSCs) and reduced failure rate of synaptic transmission after minimal-stimulation excitatory postsynaptic currents (msEPSCs). Exposure to LGI1-Ab was also associated with impaired LTP, which was however independent of Kv1.1 blockade and possibly related to reduced availability of AMPAR during LTP. However, these changes were not sufficient to cause seizures in this model. It is likely that the changes induced by the antibodies are not as severe as those induced by genetic mutation or ablation of the LGI1 gene. On the other hand, complement activation and neuronal loss may play a major role in the human and feline diseases and mouse serum has a low intrinsic complement activity (98). Further studies are needed to investigate this aspect and its relevance to the clinical phenotype.

## AMPAR-Ab Encephalitis

### Clinical Disease and *in vitro* Mechanisms

AMPAR antibodies (AMPAR-Abs) are usually associated with a typical LE, sometimes associated with extra limbic manifestations, although they can rarely present with rapidly progressive dementia or psychosis (99, 100).

AMPAR is a heterotetrameric ionotropic glutamate receptor that mediates most of the fast-excitatory transmission in the brain (101). AMPAR-Abs can be directed against the GluA1 or GluA2 subunits or both (100). Incubation of cultured rodent neurons with patients' IgG to GluA2 led to a decrease of synaptic AMPAR clusters, resulting in reduced frequency and peak amplitude of AMPAR-mediated miniature excitatory postsynaptic currents (mEPSCs) (100, 102).

### Spontaneous or Genetic Disorders

Mutations in the GluA1 or GluA2 subunits have been associated with neurodevelopmental disorders (NDs) including intellectual disability and autism (103, 104). GluA1-KO mice present impaired hippocampal synaptic plasticity (105, 106) and working memory (107–109), whereas GluA2-KO mice are hypomorphs with poor motor coordination and low explorative activity (110, 111). Conditional ablation of GluA1 or GluA2 in mice causes memory deficits and remodeling of AMPAR subunit distribution (112–115).

### Passive Transfer Models

In accordance with these findings, *in vitro* studies and *in vivo* hippocampal injection of human antibodies against the GluA2 subunit in mice was associated with synaptic downregulation of GluA2 and increased single-channel conductance in recordings of the GluA2 IgG-injected mouse, suggestive of GluA2 endocytosis and compensatory synaptic incorporation of GluA1-containing AMPARs, which have higher channel permeability (54), as observed in conditional KO models (113–115). Consistently, this compensatory increase in single-channel conductance was abrogated in KO mice deficient for GluA1 stereotactically injected with GluA2 antibodies (GluA2-Abs). Despite these compensatory mechanisms, injection of GluA2-Abs was associated with impaired LTP in the region of GluA2-IgG deposition. Both continuous icv infusion of GluA2-Abs over a 2-week period and stereotactic bilateral injections of patient IgG directly into the DG, CA1 and CA3 regions of the hippocampus, were associated with impaired memory and increased anxiety-like behavior in mice (54). Despite the observed AMPAR subunit rearrangement, mice did not show seizures. Therefore, future studies have to evaluate if these changes are associated with neuronal hyperactivity and how they are related to seizures in patients. Moreover, the pathogenicity and mechanisms associated with antibodies directed against the GluA1 subunit of AMPAR remain to be established.

## Animal Models of NDs Induced by *in utero* Exposure TO NSAbs

There has been growing interest in the possibility that maternal antibodies to neuronal antigens could cause neurodevelopmental diseases, presenting neonatally or later in life. This sprung initially from studies in mothers with MG whose babies developed arthrogryposis. The maternal antibodies were found to inhibit the function of the fetal AChR and, when crossing the placenta in the second trimester, paralyzed the babies *in utero*; consecutive pregnancies were affected (28, 116). A mouse model of maternal antibody transfer to the mouse fetus was developed to show that the maternal serum antibodies were pathogenic (36), and the model was then used to study a mother who had two consecutive children with NDs (one healthy, one with autism, and one with language disorder). The serum contained antibodies that bound to fetal cerebellar neurons in rat tissue sections and impaired motor behavior in the adult mouse offspring of injected dams (117). Since then, many studies looking for maternal antibodies in autism and testing their effects in mouse or non-human primates have been performed [see (118)], but until recently, none had defined a specific neuronal antigen that was likely to be the target of fetopathogenic antibodies.

As mentioned above, mutations in the gene encoding CASPR2 are not common but can be associated with a variety of neurological and psychiatric disorders, ranging from ASD or mental retardation and epilepsy to learning disability, schizophrenia, and Tourette syndrome (119). Mutations in the GluN genes that encode the *N*-methyl-d-aspartate (NMDA) subunits are found in children with a variety of NDs and epileptic syndromes (120). Both these proteins could be targets for antibodies that, during development, altered neurodevelopment. Table 3 summarizes the most recent work in this field.

**Table 3:** Neurodevelopmental antigens and models.

**Protein**	**Presence of antibodies in mothers of children**	**Antibodies injected and effects of antibodies on offspring of maternal-to-fetal transfer model: behavior**	**Effects of antibodies on offspring of maternal-to-fetal transfer model: neuropathology**	**References**
Acetylcholine receptor	Rare mothers with antibodies that inhibit fetal AChR, paralyze baby *in utero*, and cause multiple fixed joints, with paralysis and death *ex utero*	Maternal plasma antibodies injected into dams during E13–18 of pregnancy. Proportion of offspring who died at birth or shortly after probably due to lack of respiration	Antibodies present in mouse offspring, offspring showed fixed joints mirroring changes in human babies	(36)
CASPR2	4.9% of mothers with children diagnosed with range of motor and psychological disorders, not autism. HC 0.9%	IgG purified from plasmapheresis samples of two CASPR2-Ab-positive patients. Mice showed changes in cognition and impaired social interactions	Long-term neuropathological changes with activated microglia and glutamatergic synaptic loss	(121, 122)
CASPR2	37% of selected (brain reactive Abs) mothers of children with autism spectrum disorder; 12% of unselected women of childbearing age	MAb binding CASPR2 cloned from the mother of an autistic child. Mice showed impairments in sociability, flexible learning, and repetitive behaviors	Abnormal cortical development, decreased dendritic complexity of excitatory neurons, and reduced numbers of inhibitory neurons in the hippocampus	(123)
NMDAR (NR1 subunit)	Marginal evidence for NMDAR antibodies in mothers of children with any psychiatric/neuropsychiatric disorders	mAbs from NMDAR-Ab-positive women. Mice showed early postnatal mortality (27.2%), altered blood pH, and impaired neurodevelopmental reflexes. *Ex vivo*, NMDAR reduced in brain, with altered spontaneous excitatory postsynaptic currents. When adult, persistent hyperactivity, lower anxiety, and impaired sensorimotor gating	NMDAR was reduced (up to 49.2%), and electrophysiological properties were altered, reflected by decreased amplitudes of spontaneous excitatory postsynaptic currents in young neonates (−34.4%). Cerebellum, midbrain, brain stem volumes reduced	(124)

## Evidence For Antibodies to NSAbs in Pregnancy

### CASPR2-Abs

Only one study to our knowledge has looked for antibodies to these proteins in gestational samples from women whose children have subsequently been diagnosed with specific or non-specific neurodevelopmental conditions, comparing with mothers with no such history in their children. Coutinho et al. (121) measured a range of neuronal antibodies in Danish cohorts of early or mid-gestational sera. LGI1-Abs, AMPAR-Abs, and GABAB receptor antibodies were not found. NMDAR-Abs were not uncommon (overall 5.8%) and more common in mothers who developed psychosis at some time after the pregnancy. By contrast, CASPR2-Abs were present (4.9%) in mothers of children with a diagnosis of mental retardation or other disorders of psychological development in their children compared with only 0.9% of coded age-matched mothers with no such history. This supported the possibility that CASPR2-Abs could be a cause or contributor to neurodevelopmental diseases in the offspring. Surprisingly, CASPR2-Abs were low in mothers of autistic children and not different from the appropriate controls.

A maternal-to-fetal transfer of disease was performed in mice. The offspring of CASPR2-injected dams were normal postnatally but as adults showed changes in social interaction tests, and after termination, there was clear evidence of microglial activation and reduced glutamatergic synapses, suggesting that microglia activated by CASPR2-Abs induced changes that resulted in persistent synaptic loss (122).

A similar model was undertaken using a monoclonal CASPR2-Ab cloned from a mother of an autistic child (123). In this study, male mice exposed *in utero* to CASPR2-Abs showed an ASD-like phenotype, abnormal cortical development, and altered hippocampal neurons. Postnatal samples from selected mothers of autistic children were more often positive for CASPR2-Abs than from mothers of children with typical development or women of childbearing age. These sera were not gestational and in many cases obtained from mothers years after the affected birth.

### NMDAR-Abs

In Coutinho et al. (121), NMDAR-Abs were relatively frequent (5.8%) during pregnancy. Although NMDAR-Abs were more frequent in mothers with NDs in their children (ND mothers) than coded age- and gestation-matched mothers with no such histories (HC mothers), this difference was not significant (7.7 vs. 4.6%). Indeed, among the few reported cases of NMDARE during pregnancy, the majority of newborns were healthy, except for three cases with neurological sequelae, including neurodevelopmental delay, movement disorders, and seizures, and three cases of miscarriages and abortion (125–127). Whether these complications are due to the antibodies or to the mothers' condition severity and related pharmacological treatments during gestation is not yet clear.

Jurek et al. (124) showed a marginal increase in NMDAR-Ab titers in postnatal sera from mothers of a mixed population of neuropsychiatric disorders in a recent study, compared with mothers of unaffected children. These authors preformed a similar model of *in utero* exposure to human NMDAR-Abs, but in this case using recombinant human monoclonal NR1-reactive IgG antibodies (124). The placentally transferred antibodies bound to synaptic structures in the fetal brain, and the pups demonstrated increased mortality and transiently reduced NMDAR brain density with impaired excitatory neurotransmission. The animals displayed hyperactivity, lower anxiety, and impaired sensorimotor gaiting during adolescence and adulthood. In aged mice (10 months), the volumes of the cerebellum, midbrain, and brain stem were all reduced (124). This study suggests that prenatal exposure to NMDAR-Abs may result in children's lifelong neurodevelopmental changes that are potentially treatable and preventable, if identified in the mothers during pregnancy, although there is no evidence of that so far. Such changes might predispose to specific NDs such as autism or schizophrenia.

## Discussion and Conclusions

Animal models have helped to elucidate pathogenic mechanisms of several NSAbs. However, they often fail to recapitulate the entire phenotypic spectrum associated with human diseases. In particular, no movement disorders have been found in the models of NMDARE, and no seizures were detected in mice injected with LGI1-Abs. This could be related to several factors. Firstly, the choice of the species and strains is relevant. Nowadays, mice are the preferred animals for the majority of immune models; however, certain strains used can be resistant to development of diseases, as shown by MG models of active immunization. The gender is another potentially relevant factor, as hormones can significantly impact several immunological and neuronal aspects.

Different immunization models have different advantages and disadvantages. Intraventricular or intraparenchymal administration routes are useful in exposing the antibodies to their targets, but they may be misleading when peripheral antibodies play a major role as appears to be the case for CASPR2-Abs and LGI1-Abs. On the other hand, peripheral injection of the antibodies often requires “opening” the BBB by some method, and these methods may bias the results, allowing the antibodies to access certain brain areas and not others that are more relevant to the human disease (128, 129).

Passive transfer of antibodies is ideal to investigate the downstream mechanisms by which the patient antibodies affect their targets with possible secondary effects, but by itself, it does not appear to enlist cellular mechanisms that might be important in the human condition. Thus, it does not provide insight into the immunological mechanisms behind the generation of the antibodies nor the immunological effectors. For instance, the poor ability of human IgG to fix mouse complement is a limitation if complement activation plays a relevant part in the disease. Overall, the immune cells and the Fc receptors relevant for the human immune response might be different in animal models due to the use of alternative pathways, different effectors, and different cellular receptor affinities (130–132). Future passive transfer studies of patient-derived immune cells into humanized models or studies in non-human primates might help define the involvement of specific immune cells in the pathogenesis of these disorders.

Active immunization models could be helpful in overcoming some of these limitations and could also be more helpful in studying the effector immune mechanisms, but few studies have used this approach to date. Moreover, using peptide sequences for immunization is unlikely to generate the most appropriate pathogenic antibodies if the natural disease recognizes the native membrane protein rather than peptide or polypeptide sequences.

It is also important to note that the failure to reproduce some clinical features observed in patients might be related to the experimental approach or timing of protocols. For example, as shown for NMDAR-Ab, the presence of spontaneous seizures could be overlooked in the absence of continuous EEG monitoring (42). Similarly, antibodies may manifest their maximum effects up to 18 days after CSF infusion (38). Behavioral testing has to be carefully tailored and should take into account the effects of habituation and test repetition.

Future research and refinement of these animal models require a collaborative approach and sharing of optimal methods. Effective and reliable preclinical testing of novel treatments demands rigorous and reproducible protocols that not only allow study of the underlying neurobiology but also facilitate therapeutic studies with rapid translation to the clinic.

## Author Contributions

MG: conception and drafting of the manuscript. SW: drafting, editing, and review of the manuscript. AV: conception, drafting, editing, and review of the manuscript.

### Conflict of Interest

The University of Oxford and AV hold patents for LGI1 and CASPR2 antibody tests, licensed to Euroimmun AG. AV receives a proportion of royalties. The remaining authors declare that the research was conducted in the absence of any commercial or financial relationships that could be construed as a potential conflict of interest. The reviewer SB declared a past co-authorship with one of the authors AV to the handling Editor.
